# Identification of a novel 10 immune‐related genes signature as a prognostic biomarker panel for gastric cancer

**DOI:** 10.1002/cam4.4180

**Published:** 2021-08-12

**Authors:** Tingna Chen, Chaogang Yang, Rongzhang Dou, Bin Xiong

**Affiliations:** ^1^ Department of Gastrointestinal Surgery Zhongnan Hospital of Wuhan University Wuhan China; ^2^ Hubei Key Laboratory of Tumor Biological Behaviors Wuhan China; ^3^ Hubei Cancer Clinical Study Center Wuhan China; ^4^ The Clinical Medical Research Center of Peritoneal Cancer of Wuhan Wuhan China

**Keywords:** biomarker, gastric cancer, immune infiltration, prognostic signature, weighted correlation network analysis

## Abstract

**Background:**

Emerging evidence indicates that immune infiltrating cells in tumor microenvironment (TME) correlates with the development and progression of gastric cancer (GC). This study aimed to systematically investigate the immune‐related genes (IRGs) to develop a prognostic signature to predict the overall survival (OS) in GC.

**Method:**

The gene expression profiles of training dataset (GSE62254), validation dataset I (GSE15459), and validation dataset II (GSE84437) were retrieved from GEO and TCGA databases. In the present study, we developed a 10 IRGs prognostic signature with the combination of weighted gene co‐expression network analysis (WGCNA) and least absolute shrinkage and selection operator method (LASSO) COX model.

**Results:**

In the training dataset, the accuracy of the signature was 0.681, 0.741, and 0.72 in predicting 1, 3, and 5‐year OS separately. The signature also had good performance in validation dataset Ⅰ with the accuracy of 0.57, 0.619, and 0.694, and in validation dataset Ⅱ with the accuracy of 0.559, 0.624, and 0.585. Then, we constructed a nomogram using the signature and clinical information which had strong discrimination ability with the c‐index of 0.756. In the immune infiltration analysis, the signature was correlated with multiple immune infiltrating cells such as CD8 T cells, CD4 memory T cells, NK cells, and macrophages. Furthermore, several significant pathways were enriched in gene set enrichment analysis (GSEA) analysis, including TGF‐beta signaling pathway and Wnt signaling pathway.

**Conclusion:**

The signature of 10 IRGs we identified can effectively predict the prognosis of GC and provides new insight into discovering candidate prognostic biomarkers of GC.

## Graphical abstract

We identified a 10 immune‐related genes signature as a prognostic biomarker panel for gastric cancer. The signature was correlated with multiple immune infiltrating cells and several significant pathways.

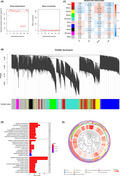

## 1 INTRODUCTION

Gastric cancer (GC) is an aggressive malignant cancer with poor prognosis and high mortality, even among patients who underwent surgical resection.^1^ According to GLOBOCAN 2018 data, GC is responsible for over 1,000,000 new cases and an estimated 783,000 deaths in 2018, making it the fifth in terms of incidence but second in terms of mortality worldwide.^2^ GC metastasizes early via lymphatic system, blood, and peritoneum, leading to recurrences within 2 years after surgery and poor long‐term survival.^3^, ^4^ Therefore, in order to improve the prognosis for GC, it is necessary to develop a new prognostic model to stratify patients with GC, thereby guiding individualized and precise treatment.

Emerging and accumulating evidence indicates that immune infiltrating cells in the tumor microenvironment (TME) play important roles in the development and progression of human cancers.^5^, ^6^, ^7^ Previously, our group systematically studied the role and mechanisms of tumor‐associated macrophages (TAMs) in TME in the development and progression of colorectal cancer (CRC). Our results found that M2‐subtype TAMs ratio was elevated at tumor invasive front, which was closely associated with aggressive phenotype and poor prognosis of CRC.^8^ Further mechanism studies revealed that M2‐subtype TAMs and CRC cells could establish "crosstalk" by secreting different cytokines to form a positive feedback loop, thereby promoting the progression and metastasis of CRC.^9^, ^10^, ^11^, ^12^ These findings indicate that TAMs play a crucial part in the development and progression of CRC. However, as a highly heterogeneous internal environment, TME was composed by complex cellular components, including not only TAMs, but also other immune infiltrating cells such as T cells, NK cells, and so on. Numerous studies have demonstrated that the prognosis of patients is affected by immune infiltrating cells, and the immune outcomes vary according to different types of cancer,^13^ such as lung tumor,^14^ breast cancer,^15^ and hepatocellular carcinoma.^16^ For GC, immune infiltrating cells have also been reported to play significant effects on tumor progression. Li and colleagues showed that M2‐subtype TAMs polarization triggered by GC‐derived mesenchymal stromal cells promoted the EMT and metastasis of GC.^17^ In addition, the accumulation of Treg cells within GC tumors underlies resistance to immune checkpoint blockade (ICB).^18^ However, the prognostic value of immune infiltration in GC still needs further investigation based on comprehensive analysis and large sample statistics.

Nowadays, computational methods have been developed based on gene expression profiles, which provide effective tools for systematic analysis to identify candidate biomarkers.^19^, ^20^ For the first time, we identified a novel prognostic signature panel using the combination of WGCNA and LASSO‐Cox model in GC based on immune‐related genes (IRGs). The signature could effectively predict the overall survival (OS) of GC patients and stratify patients into subgroups according to risk score (RS). The efficacy of the signature was validated in two external datasets. Then, we constructed a nomogram with the combination of the signature and clinical features in order to improve clinical decisions. Finally, we explored the underlying mechanisms by analyzing the relationship between immune infiltration and the signature, along with assessing significant pathways enriched in high RS and low RS groups. Our findings provide a model for patient classification and individualized treatment.

## 2 MATERIALS AND METHODS

### 2.1 Data collection and preprocessing

**FIGURE 1:**
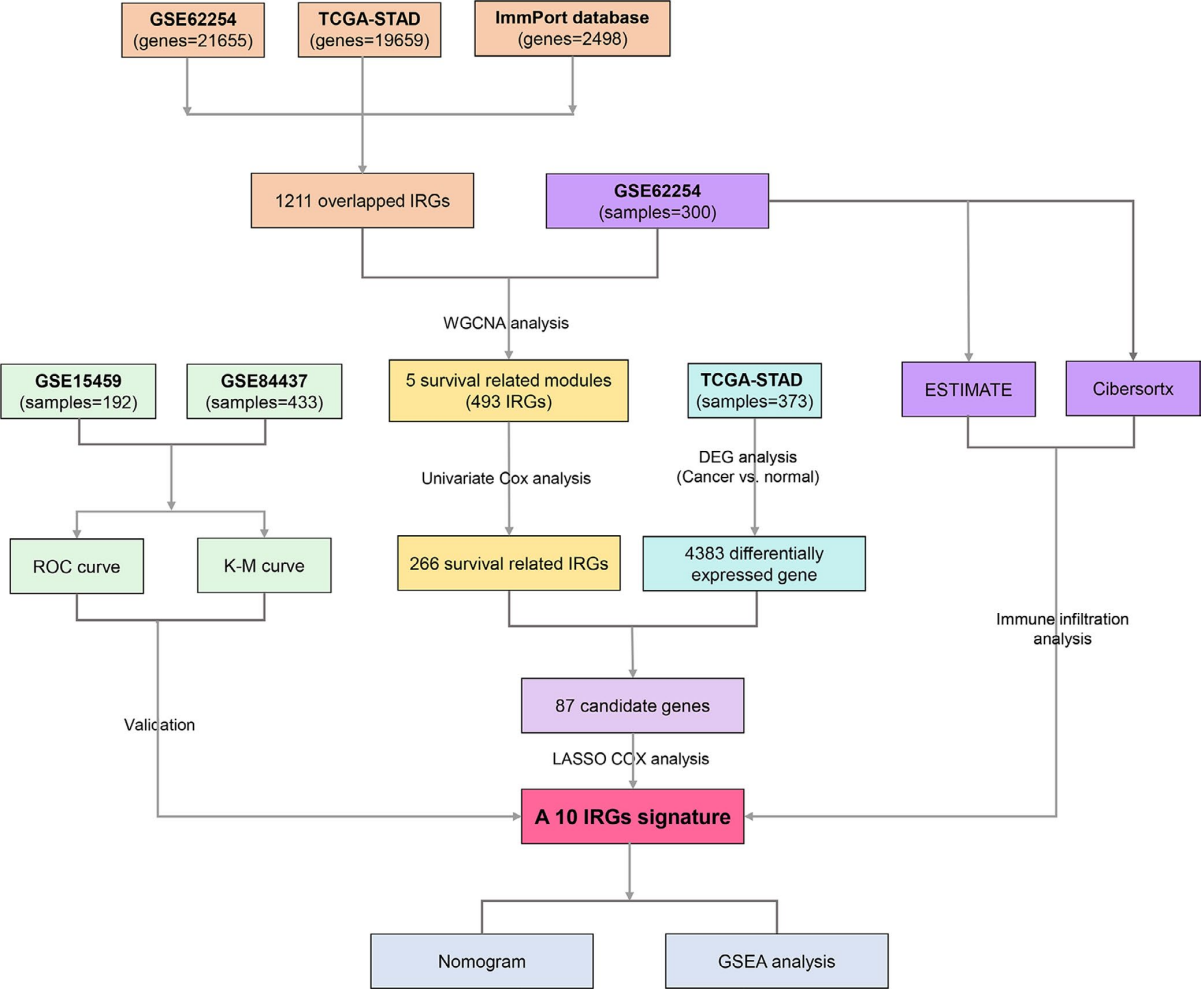
Flow diagram of the study. Data processing, analysis, and validation are shown in the picture

A flow diagram of the data preparation, processing, analysis, and validation is shown in Figure 1. The raw data of the training dataset GSE62254(samples = 300) were downloaded from the Gene Expression Omnibus database (GEO: http://www.ncbi.nlm.nih.gov/geo/) and were further normalized by Robust multi‐array average (RMA) using R package “affy”.^21^ The probes were concerted to gene symbols according to the platform GPL570 (Affymetrix Human Genome U133 Plus 2.0 Array). The validation dataset Ⅰ GSE15459 (samples = 192) which was also based on platform GPL570 was acquired and processed in the same way. The validation data Ⅱ GSE84437 (*n* = 433) which was based on platform GPL6947 was log2 transformed and normalized using R package “limma.” The stomach adenocarcinoma (STAD) RNA‐seq read counts data were retrieved from The Cancer Genome Atlas database (TCGA, https://portal.gdc.cancer.gov/) and were divided into cancer group (samples = 342) and normal group (samples = 30), which were normalized and selected for differentially expressed gene (DEG) analysis using R package “DESeq2”.^22^ The immune‐related genes (IRGs) were derived from the ImmPort database (https://immport.niaid.nih.gov/). In total, 1211 overlapped genes from the GSE62254 and TCGA‐STAD datasets and the IRGs were filtered for further analysis. The Venn diagram of overlapped IRGs was plotted using R package “VennDiagram” (https://CRAN.R‐project.org/package=VennDiagram). All analyses were carried out by R version 3.6.1.

### 2.2 Identification of prognostic genes by weighted gene co‐expression network

The training dataset GSE62254 was used to construct the weighted gene co‐expression network analysis (WGCNA) using “WGCNA” package according to the protocol in R software.^19^ Briefly, a similarity matrix between un‐signed gene expression profiles was constructed based on pairwise Pearson correlation. Then, the similarity matrix was converted to an adjacency matrix using a power adjacency function. The power was chosen based on the scale‐free topology criterion according to the scale‐free topology index (R2) as 0.9.^23^ Next, the adjacency matrix was transformed into a topological overlap matrix (TOM) to detect modules.^24^ The modules were cut using the dynamic tree cut algorithm.^24^ We cut the genes into modules by [blockwiseModules] method with following parameters: minModuleSize = 20, mergeCutHeight = 0.2, deepSplit = 2, and verbose = 3.

To extract co‐expressed genes most related to OS for further analysis, the modules and external clinical traits were related by calculating the module eigengenes (MES), which were the representatives of all genes in a module. The modules with *p *< 0.05 were selected as survival‐correlated modules.

For each gene in the modules of interest, the survival difference was analyzed by univariate Cox regression analysis using R package “survival”.^25^ Survival‐related genes were identified with *p* < 0.05. Subsequently, Gene Ontology (GO) analysis and Kyoto Encyclopedia of Genes and Genomes (KEGG) pathways analysis of the survival‐related gene set were performed using R package “clusterProfiler”.^26^

### 2.3 Identification of DEGs and construction of a prognostic classifier

Differentially expressed genes (DEGs) between cancer samples and normal samples in TCGA‐STAD were identified using R package “deseq2”.^22^ DEGs need to satisfy the following criterions: log_2_|fold change (FC)|≥1 and *p* < 0.05. DEGs were visualized using R package “pheatmap” (https://CRAN.R‐project.org/package=pheatmap). The intersection of survival‐related genes and DEGs was chosen as candidate genes for the construction of the classifier.

LASSO is a method to reduce the estimation variances in high‐dimensional predictors.^20^ To screen out the most representative prognostic biomarkers, the expression data of the candidate genes were integrated into the LASSO regression by R package “glmnet”.^27^ After selection of the key genes influencing the OS of the patients, the multivariate Cox regression analysis was performed to attain the coefficients of the genes using R package “survival”.^28^ Then the RS of each sample was calculated using the formula: RS = Σ (Coef of gene*Expression level of gene). The samples were divided into high RS group and low RS group with the median cut‐off of RS.

### 2.4 Verification of the signature

The RS of the GSE15459 dataset and the GSE84437 dataset was calculated using the formula above. Time‐dependent ROC curve and calibration plot in 1, 3, and 5 years were used to assess the accuracy of the signature. Samples were divided into high RS group and low RS group according to the median cut‐off. Kaplan–Meier plot was performed to explore the prognostic difference between high RS and low RS groups.

### 2.5 Construction of nomogram

Nomogram is a device to predict the prognosis for clinical convenience.^29^ The index of concordance (C‐index) and calibration plot were used to assess the discrimination ability of the nomogram using a bootstrap manner under 1000 resampling. The construction and validation of the nomogram were through R package “rms” (https://CRAN.R‐project.org/package=rms). The area under the curve (AUC) of the ROC curve was calculated to detect the accuracy of the nomogram using R package “timeROC”.^30^

### 2.6 Estimation of immune infiltration

The gene expression data from GSE62254 were adopted to explore the relationship between the high and low RS groups. First, we applied ESTIMATE (Estimation of Stromal and Immune cells in MAlignant Tumor tissues using Expression data) algorithm to calculate the immune score and the stromal score of the samples by R package “estimate”.^31^ The correlation curve was plotted to see if there was relationship between OS and immune or stromal score by R package “ggpubr” (https://CRAN.R‐project.org/package=ggpubr). Subsequently, we uploaded the expression data to CIBERSORTx (Cell type Identification By Estimating Relative Subsets Of RNA Transcripts, https://cibersortx.stanford.edu/runcibersortx.php) to calculate immune cell type fractions based on the default signature matrix at 100 permutations.^32^ The visual display of the relationship between immune infiltration and the signature was presented via R package “vioplot” (https://github.com/TomKellyGenetics/vioplot), and the relationship between immune infiltrating cells was presented via R package “corrplot” (https://github.com/taiyun/corrplot).

### 2.7 Gene set enrichment analysis (GSEA)

GSEA analysis (http://software.broadinstitute.org/gsea/index.jsp) was applied to identify the potential functions of the signature in dataset GSE62254 between high RS and low RS groups.^33^ The whole genome of RNA‐seq data in GSE62254 was adopted as gene list and the high and low RS groups were used as the phenotype labels. The metric for ranking genes parameter was Signal2Noise. The reference gene set was “c2.cp.kegg.v6.2.symbols.gmt.” The number of permutations was 1000. We selected an ordered list of significant enriched pathways with nominal *p* < 0.01 and false discovery rate (FDR) <25%.

## 3 RESULTS

### 3.1 Construction of weighted gene co‐expression network and identification of survival relevant modules

**FIGURE 2:**
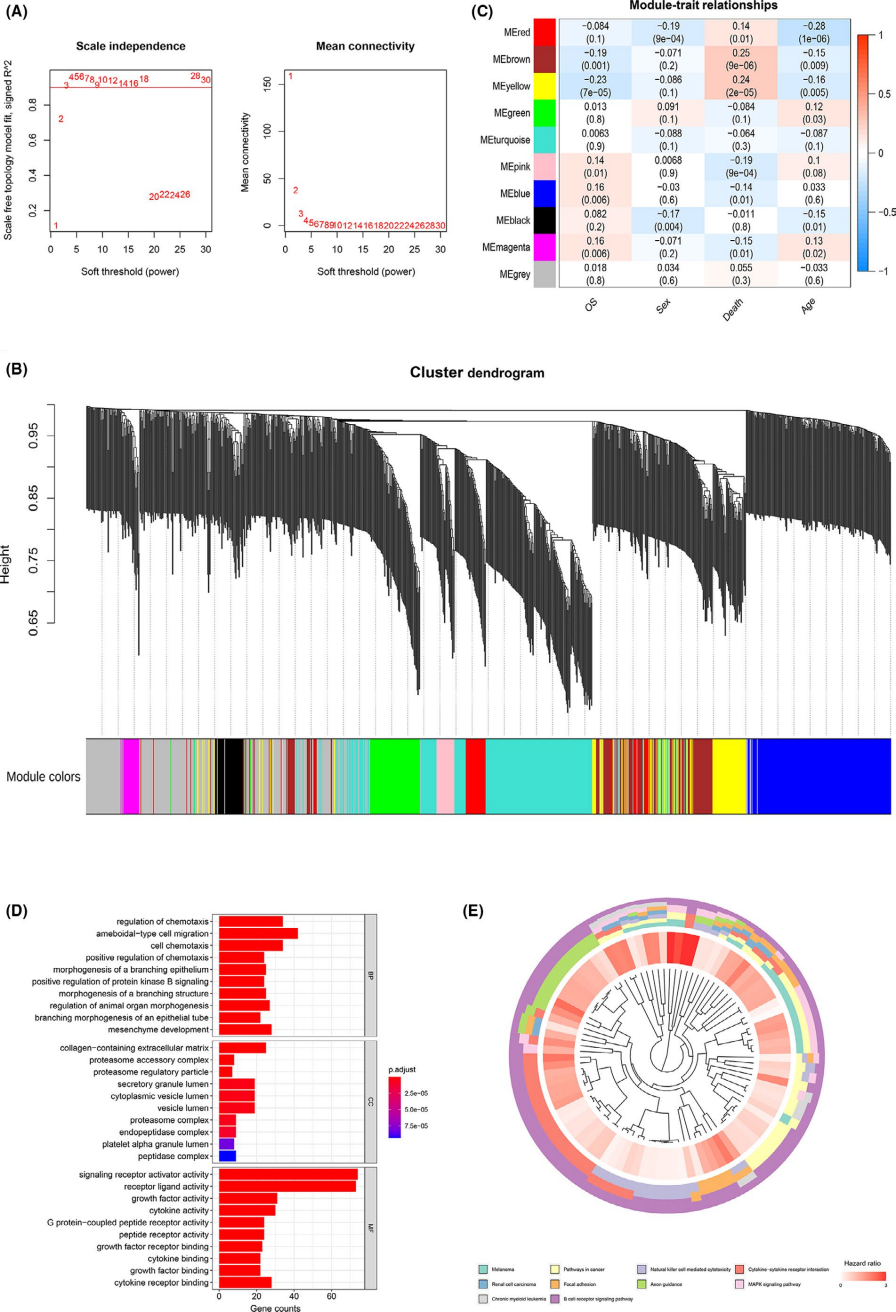
Overlapping IRGs were analyzed by WGCNA. (A) The scale‐free fit index for soft‐thresholding powers. Left: the relationship between the soft‐threshold and scale‐free R2. Right: the relationship between the soft‐threshold and mean connectivity. Different modules are labeled in different colors. (B) Dendrogram of differentially expressed genes clustered in the training dataset. (C) Heatmap of the correlation between module eigengenes and different clinical information of the GSE dataset (OS, Sex, Death, and Age). (D) GO analysis of survival‐correlated genes in yellow module. (E), KEGG pathway analysis of survival‐correlated genes in yellow module

Based on 1211 overlapping IRGs (Figure S1), the co‐expression network was constructed using the WGCNA approach. The power 3 was chosen based on a scale‐free R^2^ (*R* ^2 ^= 0.9, Figure 2A). Nine modules were identified (Figure 2B). Then, the relationship between modules and clinical traits was explored (Figure 2C). The clinical information of interest includes: OS, sex, death, and age. Results showed that five modules correlated with OS (MEbrown: *r* = −0.19, *p *= 0.001; MEyellow: *r* = −0.23, *p *= 7e‐05; MEpink: *r* = 0.14, *p *= 0.01; MEblue: *r* = 0.16, *p *= 0.006; MEmagenta: *r* = 0.16, *p *= 0.006) Therefore, we chose all five modules for further analysis. By applying univariate Cox regression analysis to all IRGs in the yellow module, 266 genes were identified of prognostic value (*p *< 0.05, Table S1).

Functional analysis of these genes was performed by GO analysis (Figure 2D, Table S2) and KEGG pathway analysis (Figure 2E, Table S3). The biological processes were enriched in regulation of chemotaxis, ameboidal‐type cell migration, and cell chemotaxis. The cellular components were enriched in collagen‐containing extracellular matrix, proteasome accessory complex, and proteasome regulatory particle. The molecular functions were enriched in signaling receptor activator activity, receptor ligand activity, and growth factor activity. KEGG pathway analysis showed that the top 10 significant pathways were Melanoma, pathways in cancer, natural killer cell‐mediated cytotoxicity, cytokine–cytokine receptor interaction, renal cell carcinoma, focal adhesion, axon guidance, MAPK signaling pathway, chronic myeloid leukemia, and B‐cell receptor signaling pathway.

### 3.2 Construction of the prognostic classifier

**FIGURE 3:**
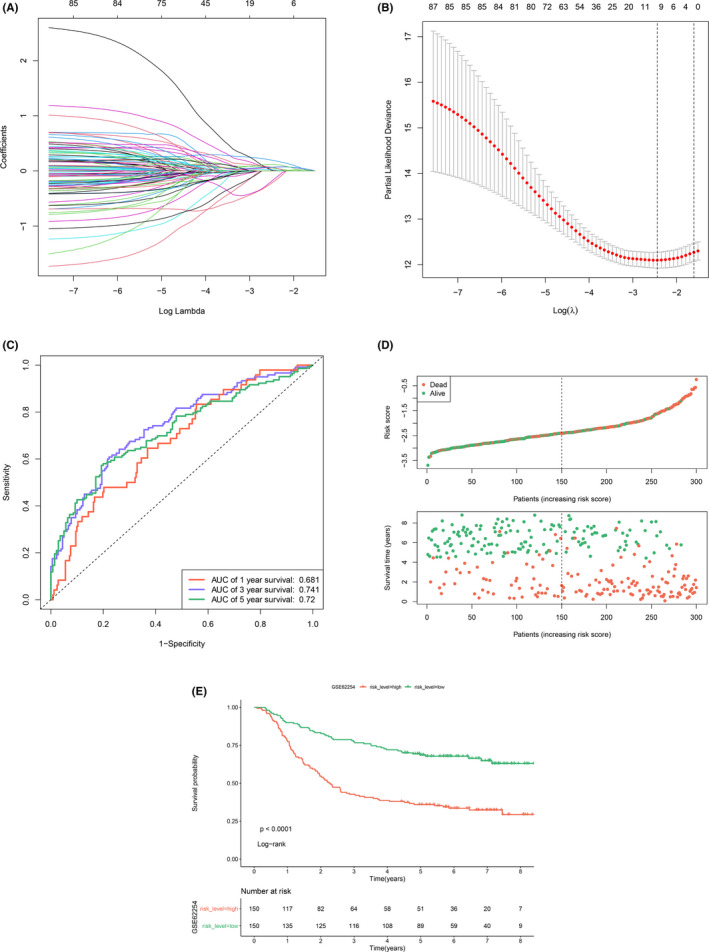
Identification of the signature and stratification of patients using RS. (A) least absolute shrinkage and selection operator method (LASSO) coefficient profiles of the 87 prognosis‐correlated genes. (B) Determination of the number of the components of the classifier. The vertical lines are plotted according to the minimum criteria and 1‐standard error criterion. The left vertical line represents the 10 IRGs finally identified. (C) The time‐dependent ROC curve predicting the 1‐year, 3‐year, and 5‐year survival rates. (D) Distribution of RS and the relationship between OS and RS. The high‐risk and low‐risk groups were stratified at optimal cut‐off calculated by X‐tile software. (E) Kaplan–Meier curve showing OS in high RS and low RS groups

To construct a stable model specific for GC, 87 candidate genes to build the classifier were obtained from the intersection of 4383 DEGs in TCGA‐STAD and 266 survival‐related genes. The visualization of DEG analysis of TCGA‐STAD was represented in volcano plot (Figure S2A) and heatmap (Figure S2B). Using the expression data of 87 candidate genes in GSE62254, the LASSO analysis screened out 10 genes (BMPR1B, GHR, IL11RA, INHBB, NPR3, OBP2A, PTN, R3HDML, TAC1, and TPM2) as the most representative genes to construct the signature (Figure 3A,B). Multivariate Cox regression analysis of the 10 IRGs was carried out to explore the relationship of each gene and OS (Table 1), which indicated that 2 IRGs were protective factors including OBP2A and R3HDML, while 8 IRGs were risk factors including BMPR1B, GHR, IL11RA, INHBB, NPR3, PTN, TAC1, and TPM2. The RS of each patient was computed according to the Cox regression coefficient and the expression value of each gene using the formula: RS = (0.12784 × BMPR1B expression) + (0.02741 × GHR expression) + (0.3035 × IL11RA expression) + (0.34105×INHBB expression) + (0.15621 × NPR3 expression) + (−0.84737 × OBP2A expression) + (0.0486 × PTN expression) + (−0.78119 × R3HDML expression) + (0.03729 × TAC1 expression) + (0.17652 × TPM2 expression).

**TABLE 1:** Information for 10 IRGs signature screened by LASSO Cox regression

Symbol	Coefficient	*p* value	HR	Low 95% CI	High 95% CI
BMPR1B	0.12784	0.3497	1.1364	0.8693	1.486
GHR	0.02741	0.8168	1.0278	0.8151	1.296
IL11RA	0.3035	0.382	1.3546	0.6859	2.675
INHBB	0.34105	9.75E−05	1.4064	1.1847	1.67
NPR3	0.15621	0.5495	1.1691	0.701	1.95
OBP2A	−0.84737	0.1554	0.4285	0.1331	1.38
PTN	0.0486	0.7633	1.0498	0.7651	1.44
R3HDML	−0.78119	0.0834	0.4579	0.1891	1.109
TAC1	0.03729	0.5914	1.038	0.9058	1.189
TPM2	0.17652	0.5758	1.1931	0.6428	2.214

In order to assess the prediction ability of RS, tROC curve was plotted and the results showed that RS could effectively predict 1, 3, and 5‐year OS of the training dataset with the AUC of 0.681, 0.741, and 0.72, respectively (Figure 3C). We presented the distribution of RS and samples’ survival status in Figure 3D. The Kaplan–Meier analysis showed that the high RS group represented worse prognosis (*p *< 0.0001, Figure 3E).

### 3.3 Verification in two external datasets

**FIGURE 4:**
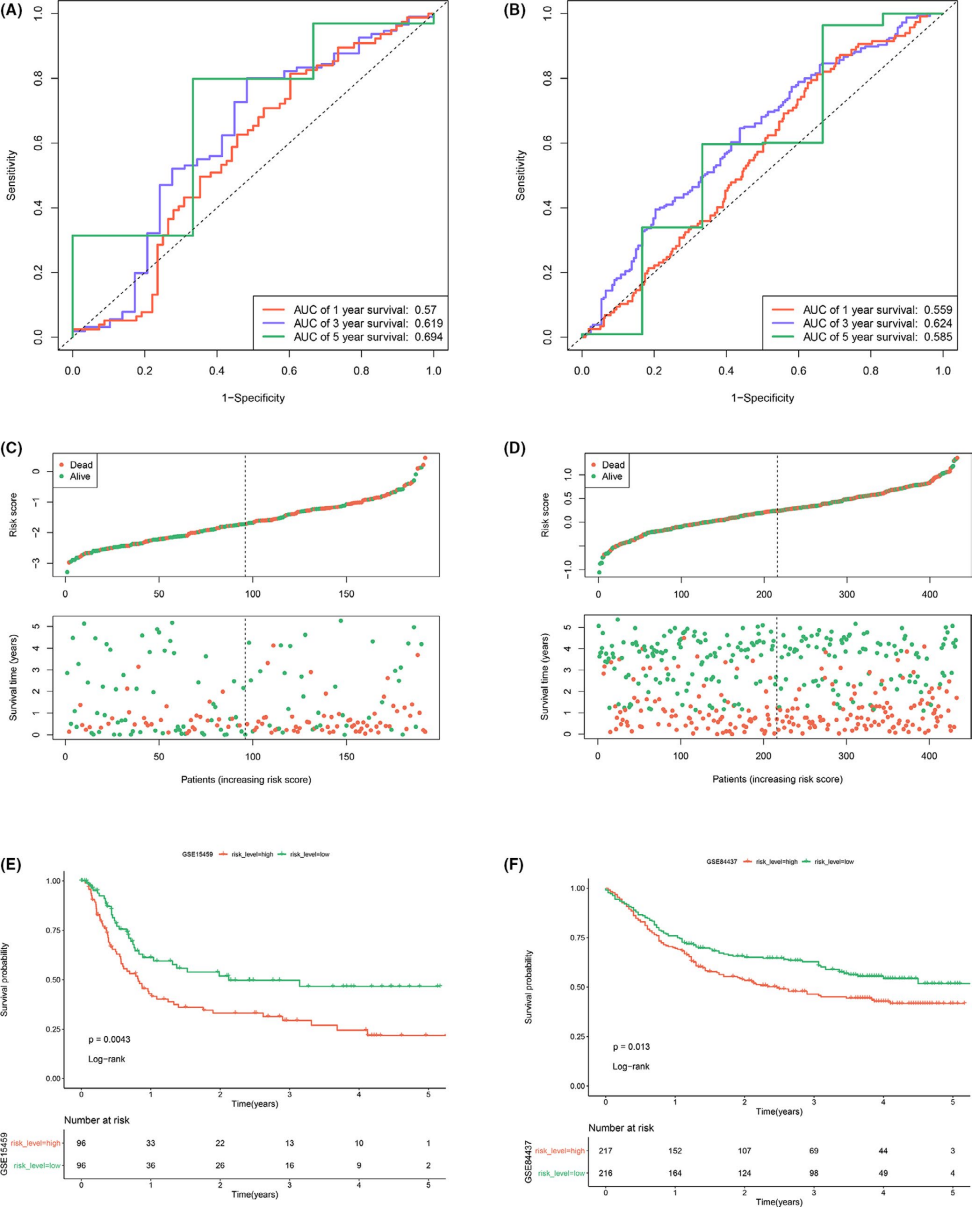
Validation of the signature in two external datasets. (A) The time‐dependent ROC curve predicting the 1‐year, 3‐year, and 5‐year survival rates of GSE15459. (B) The time‐dependent ROC curve predicting the 1‐year, 3‐year, and 5‐year survival rates of GSE84437. (C) Distribution of RS and the relationship between OS and RS in GSE15459. (D) Distribution of RS and the relationship between OS and RS in GSE84437. (E) Kaplan–Meier curve analysis between the high RS and low RS groups of GSE15459. (F) Kaplan–Meier curve analysis between the high RS and low RS groups of GSE84437

The RS was calculated as the formula above in GSE15459 and GSE84437 for the validation. Time‐dependent ROC curve showed that the accuracy of predicting 1, 3, and 5‐year OS in GSE15459 was 0.57, 0.619, and 0.694 (Figure 4A), and the accuracy in GSE84437 was 0.559, 0.624, and 0.585 (Figure 4B). All samples in the two validation datasets were divided into two groups using the median cut‐off in each dataset. The distribution of RS and the relationship between OS and RS in the datasets are shown in Figure 4C,D. The Kaplan–Meier analysis showed that the high RS group had significantly worse OS in GSE15459 (*p *= 0.0043, Figure 4E) and GSE84437 (*p *= 0.013, Figure 4F). The univariate and multivariate Cox regression analyses showed that the 10 IRGs classifier was an independent prognostic factor in the training dataset and the two validation datasets (Table 2). The expression of 10 IRGs in high RS and low RS groups showed that 8 risk factors expressed higher in the high RS group, while 2 protective factors expressed higher in the low RS group in the training dataset, the TCGA dataset, and the two validation datasets (Figure S3).

**TABLE 2:** Univariate and multivariate Cox regression analysis of clinical information and RS

Univariate Cox regression analysis			Multivariate Cox regression analysis		
Training dataset					
Risk factors	HR (95%CI)	*p* value	Risk factors	HR (95%CI)	*p* value
Gender (male vs. female)	0.90(0.65–1.27)	0.559	Gender (male vs. female)	1.07(0.75–1.52)	0.716
Age (≥60 vs. <60)	1.24(0.88–1.74)	0.217	Age (≥60 vs. <60)	2.10(1.46–3.00)	<0.001
Stage	3.41(2.34–4.96)	<0.001	Stage	1.32(0.89–1.96)	0.175
RS	2.72(2.15–3.44)	<0.001	RS	2.76(2.13–3.58)	<0.001
Validation dataset I					
Risk factors	HR (95%CI)	*p* value	Risk factors	HR (95%CI)	*p* value
Gender (male vs. female)	1.4(0.91–2.17)	0.127	Gender (male vs. female)	0.76(0.48–1.21)	0.542
Age (≥60 vs. <60)	0.98(0.64–1.51)	0.936	Age (≥60 vs. <60)	1.02(1.00–1.03)	0.07
Stage	2.79(2.14–3.64)	<0.001	Stage	3.07(2.31–4.07)	<0.001
RS	1.39(1.08–1.79)	0.011	RS	1.58(1.21–2.08)	<0.001
Validation dataset II					
Risk factors	HR (95%CI)	*p* value	Risk factors	HR (95%CI)	*p* value
Gender (male vs. female)	1.26(0.93–1.70)	0.141	Gender (male vs. female)	1.24(0.91–1.67)	0.171
Age (≥60 vs. <60)	1.79(2.34–2.39)	<0.001	Age (≥60 vs. <60)	1.94(1.45–2.61)	<0.001
RS	1.54(1.14–2.07)	0.005	RS	1.72 (1.27–2.33)	<0.001

### 3.4 Development and assessment of the nomogram

**FIGURE 5:**
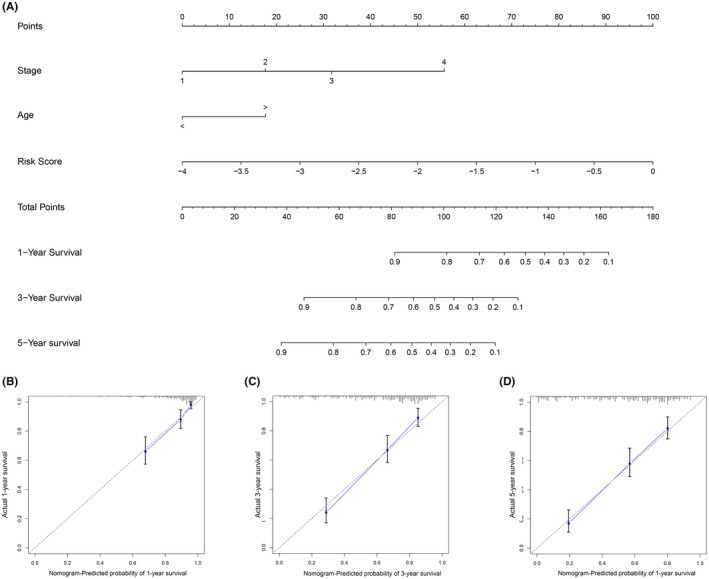
Construction of the nomogram. (A) The nomogram for predicting survival possibilities. (B–D), The calibration plot of the nomogram for predicting 1‐year, 3‐year, and 5‐year survival rates

For the convenience of clinical practice, we developed a nomogram of RS and clinical information using the training dataset GSE62254 (Figure 5A). Instructions: Draw a straight line upward from each IRG and sum all IRG’s points to attain total points, then draw a straight line downward from the “Total Points” axis to find the patients’ survival possibility in 1, 3, and 5 years. The c‐index was 0.7555135 which indicated that the model had good discrimination ability. Figure 5B–D shows that the nomogram showed good performance on account that the calibration plots were close to the 45° line.

### 3.5 Immune infiltration analysis

**FIGURE 6:**
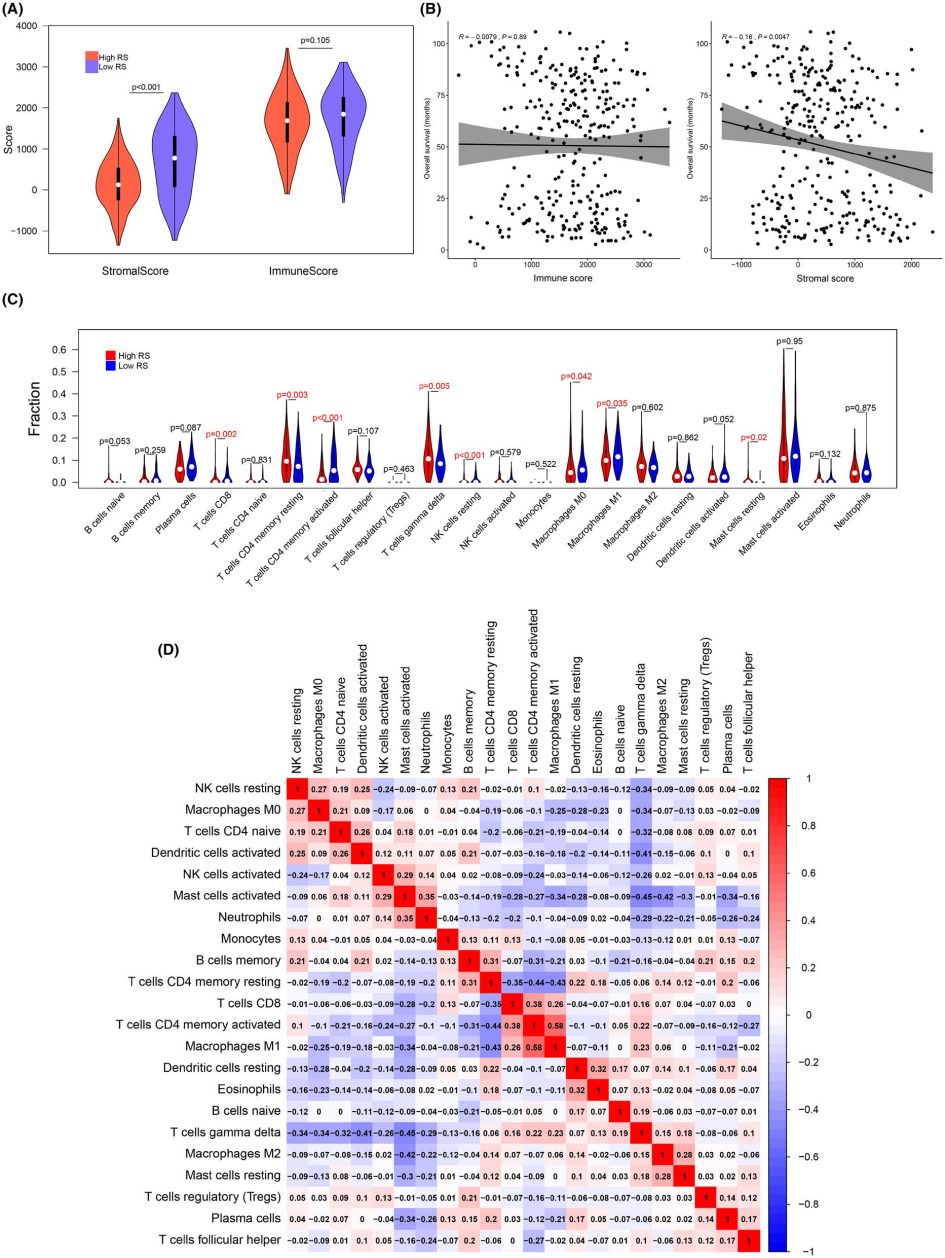
Immune infiltration analysis. (A) The violin plot of the difference in stromal score and the immune score between high RS and low RS groups. (B), The correlation plots of the relationship of OS and the stromal score, along with the relationship of OS and the immune score. (C) The violin plot showed the difference in 22 immune infiltrating cells between high RS and low RS groups. (D) The correlation plots of the nine immune infiltrating cells related to RS

The difference between high and low RS groups was significant in the stromal score (*p *< 0.001), while no difference is shown in the immune score (Figure 6A), which indicated that the overall level of immune infiltration was similar among GC patients. The negative correlation was observed between the stromal score and OS in the correlation plot (*r* = −0.16, *p *= 0.0047, Figure 6B).

Since the 22 subsets of leukocyte were reported to hold promising prognostic value in cancers,^34^ to investigate if the imbalance of these infiltrating immune cells were correlated with the signature we identified and OS, the expression profiles were further uploaded to CIBERSORTx to compute the fractions of the 22 immune infiltrating cells.

The differences of 22 infiltrating immune cells were analyzed between high and low RS groups (Figure 6C). Eight subtypes of infiltrating cells showed difference between high RS and low RS groups (*p *< 0.05). Five cell fractions were downregulated in the high RS group including T cells CD8 (*p *= 0.002), T cells CD4 memory activated (*p *< 0.001), NK cells resting (*p *< 0.01), Macrophages M0 (*p *= 0.042), and Macrophages M1 (*p *= 0.035), while three cell fractions were upregulated in the high RS group including, T cells CD4 memory resting (*p *= 0.003), T cells gamma delta (*p *< 0.01), and Mast cells resting (*p *= 0.02).

The Pearson correlation was applied to explore the relationship between infiltrating immune cells and OS (Table S4, Figure S4). The positive correlation was observed in three cell fractions including T cells CD4 memory activated (*r* = 0.16, *p *= 0.0048), Plasma cells (*r* = 0.13, *p *= 0.022), and NK cells resting (*r* = 0.11, *p *= 0.048), while negative correlation was observed in Macrophages M2 (*r* = −0.12, *p *= 0.032). Figure 6D shows the correlations between 22 immune infiltrating cells, which indicated that there were weakly relationships between those cell types.

### 3.6 GSEA analysis of potential pathways

**FIGURE 7:**
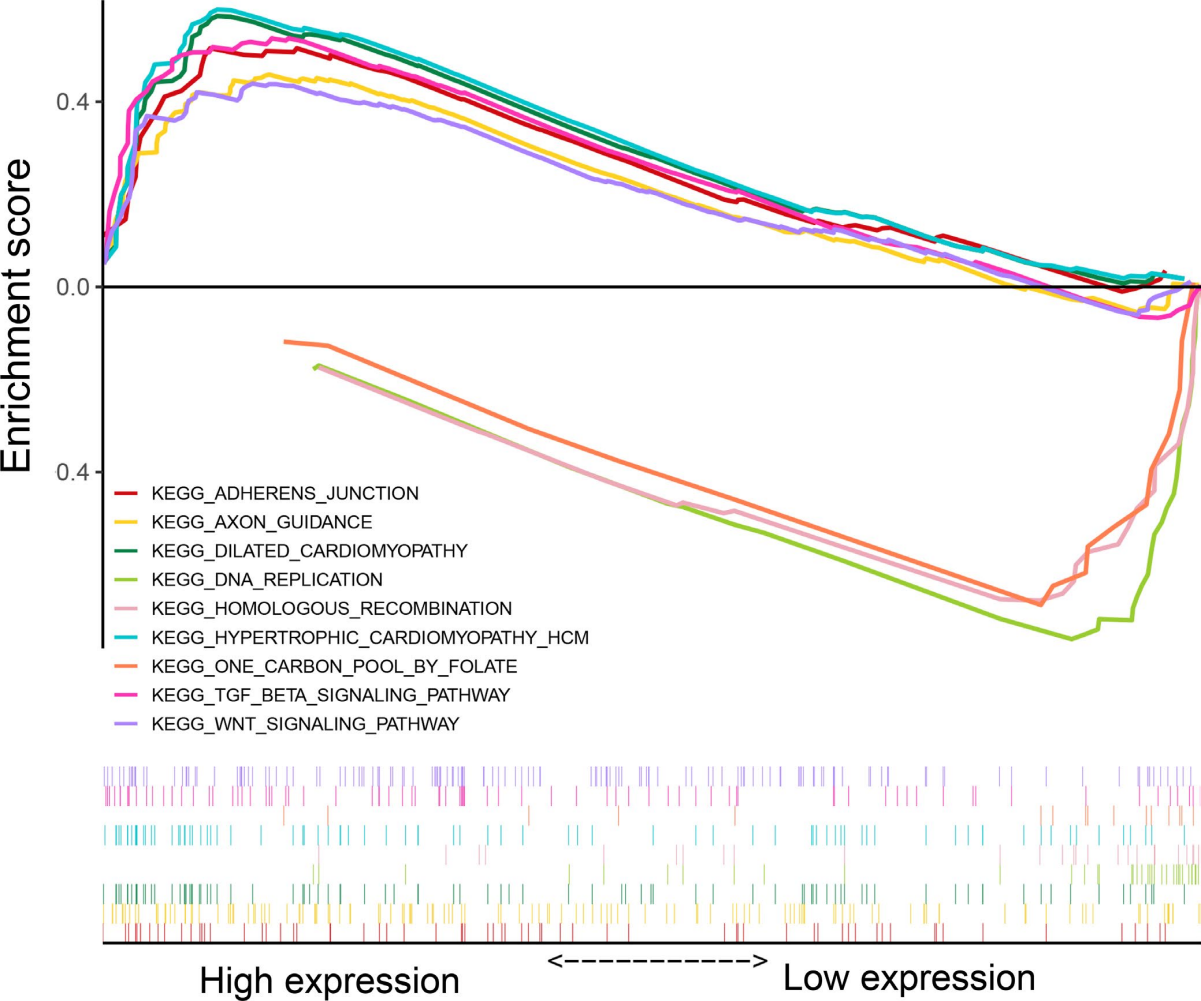
Results of the gene set enrichment analysis (GSEA) analysis

There were six pathways enriched in the high RS group and three pathways enriched in the low RS group (Figure 7). The pathways enriched in the high RS group were axon guidance (nominal *p *= 0.004, FDR = 0.196, NES = 1.58, gene size = 128), adherens junction (nominal *p* = 0.008, FDR = 0.248, NES = 1.67, gene size = 66), dilated cardiomyopathy (nominal *p* = 0.008, FDR = 0.162, NES = 1.66, gene size = 88), hypertrophic cardiomyopathy HCM (nominal *p* = 0.008, FDR = 0.221, NES = 1.66, gene size = 1.66), WNT signaling (nominal *p* = 0.008, FDR = 0.205, NES = 1.56, gene size = 146), and TGF‐beta signaling pathway (nominal *p* = 0.0097, FDR = 0.179, NES = 1.60, gene size = 81). The pathways enriched in the low RS group were one carbon pool by folate (nominal *p *< 0.001, FDR = 0.123, NES = −1.85, gene size = 16), homologous recombination (nominal *p* = 0.006, FDR = 0.136, NES = −1.74, gene size = 26), and DNA replication (nominal *p* = 0.009, FDR = 0.112, NES = −1.73, gene size = 36).

## 4 DISCUSSION

With the development of transcriptome analysis methods such as quantitative PCR and microarray, the molecular signatures predicting clinical outcomes have been developed in GC and several approaches were adopted to distinguish the subtypes of GC based on gene expression.^35^, ^36^, ^37^, ^38^ However, these researches were genome‐wide, which was inconvenient for clinical practice. Recently, signatures derived from the whole genome to predict the long time survival of GC patients consisting of a few genes were invented to improve the clinical management.^39^, ^40^, ^41^, ^42^ Nevertheless, the genes involved in the classifiers did not overlap, probably because of the high heterogeneity of GC. There is still development and improvement space for new signatures.

Previous studies demonstrated that immune features significantly affected the survival of GC patients.^38^, ^43^ Additionally, a signature of 14 immune‐related gene pairs based on 25 genes was established by defining a gene pair that the expression of one gene was stably higher than the other,^44^ but the biological connection of genes in a gene pair was uncertain and they used univariate Kaplan–Meier curve analysis to screen out survival‐related genes to put in LASSO analysis which was only suitable for dichotomous outcomes. After construction of the classifier, they only used Kaplan–Meier curve to evaluate the model. In our study, we applied WGCNA analysis to all IRGs to identify co‐expressed gene modules and subsequently correlated these modules with clinical traits. The signature in this paper was built based on the IRGs in the interested co‐expressed gene module. To screen out the survival‐related genes, univariate Cox regression analysis was adopted, which was suitable for continuous variables as gene expression profiles. Furthermore, we constructed a nomogram for clinical convenience and used time‐dependent ROC curve and calibration plot for evaluation.

BMPR1B, GHR, IL11RA, INHBB, NPR3, OBP2A, PTN, R3HDML, TAC1, and TPM2 were used to build the signature. Previous studies stated the correlation between some of these IRGs and GC. GHR regulates GC cell growth and apoptosis through controlling G1 cell cycle progression via PI3K/AKT signaling pathway.^45^ IL11RA was reported to have common copy number alteration in GC cell lines.^46^ Tachykinin‐1 (TAC1) is centrally involved in gastric secretion, motility, mucosal immunity, and cell proliferation and was silenced by promoter hypermethylation in GC.^47^ miRNA‑183‑5p.1 restricts TPM1 (tropomyosin 1), TPM2, and TPM3 and promotes cell proliferation, migration, and invasion.^48^

For the better understanding of the RS and the immune infiltration, we applied the ESTIMATE algorithm and utilized the CIBERSORTx web tool. A previous study revealed the stromal score predicted poor prognosis in GC.^49^ The stromal score was higher in the high RS group, which indicated that stromal changes in the progression of GC might be hazardous. CD4+ T‐cell response was reported to be harnessed to mediate the regression of a metastatic epithelial cancer.^50^ The latest research shows that CD4+ T cells augment immune‐mediated elimination of tumors.^51^ In our analysis, CD4 memory T cells activated were distinctly downregulated in high RS group compared to low RS group and were notably correlated with RS, which might provide information for further experiment on treatment of GC. Our study also found that macrophages M1 were significantly downregulated in high RS group and negatively correlated with RS. Macrophages M1 inhibit, whereas macrophages M2 promote gastric tumor progression.^52^ The strategies reconverting M2 to M1 were proposed in anti‐cancer treatment.^53^, ^54^ The treatments targeting immune infiltrating cells are worth continuing excavation.

Nine pathways were differentially enriched between high RS and low RS groups by GSEA analysis. TGF‐beta shapes the TME to restrain anti‐tumor immunity by restricting T‐cell infiltration.^55^ WNT signaling‐targeted therapeutics combined with immune checkpoint blockers might be applicable to treat cancers with immune invasion,^56^ and WNT inhibitors could be effective anti‐metastatic drugs for GC.^57^

However, there are still limitations in the present research. First, our research is retrospective and a prospective validation cohort is needed. Additionally, the number of IRGs detected on different platforms was different and not complete which meant several genes were not involved in the analysis. Finally, the underlying mechanism of the signature genes was not fully identified and further experimental studies are needed for the better understanding of their functions.

## 5 CONCLUSIONS

In summary, we successfully developed and validated a 10 IRGs signature which could effectively predict the OS of GC patients. The signature was also correlated with multiple types of immune infiltrating cells and significant pathways. In addition, a nomogram based on these IRGs was constructed for clinical convenience. Therefore, our study could provide information for further immune‐related work and precise immunotherapy in GC.

## CONFLICT OF INTEREST

No conflict of interest existed in this study.

## ETHICAL APPROVAL

The ethical approval was not sought from an institutional review board or ethics committee prior to commencing this study. This paper was bioinformatics analysis and did not require ethical approval.

## Supporting information

Table S1‐S4‐Fig S1‐S4

## Data Availability

The data that support the findings of this study are openly available in ImmPort database (https://immport.niaid.nih.gov/ ), Gene Expression Omnibus (GEO) database (GSE62254, GSE15459, GSE84437), and The Cancer Genome Atlas (TCGA) database (https://genomecancer.ucsc.edu/).
