# Supplementary material for: Identification of a novel 10 immune‐related genes signature as a prognostic biomarker panel for gastric cancer
Source: Cancer Med. 2021 Aug 12;10(18):6546–60. doi: 10.1002/cam4.4180 (PMC8446556; doi:10.1002/cam4.4180)
Supplement: Supplementary file 1 — Table S1‐S4‐Fig S1‐S4 [file CAM4-10-6546-s001.pdf]

Table S1 Univariate Cox regression analysis results of 266 survival-related genes

IRGs	HR	Low 95% CI	High 95% CI	P value
TAC1	1.290696	1.19279	1.396638	2.30E-10
TNFRSF11A	0.648643	0.56513	0.744497	7.48E-10
SLIT2	1.385918	1.244223	1.54375	3.01E-09
GHR	1.379461	1.239125	1.53569	4.18E-09
DES	1.609925	1.37227	1.888737	5.11E-09
ANGPTL1	1.332518	1.203983	1.474774	2.91E-08
AGTR1	1.398752	1.242092	1.575172	3.07E-08
VIP	1.25402	1.155473	1.360971	5.94E-08
PGR	2.125104	1.617222	2.792485	6.31E-08
PENK	1.688485	1.395895	2.042404	6.84E-08
TPM2	2.859857	1.947431	4.199779	8.34E-08
PTN	1.65026	1.364996	1.995141	2.30E-07
NOV	1.549384	1.311579	1.830305	2.60E-07
SHC4	2.122328	1.587619	2.837125	3.75E-07
LTBP1	1.52391	1.29406	1.794585	4.41E-07
NPR3	2.48413	1.7434	3.539579	4.74E-07
INHBB	1.456562	1.255066	1.690408	7.40E-07
BMPR1B	1.606132	1.330843	1.938367	7.84E-07
PSMB8	0.625717	0.519329	0.7539	8.18E-07
KCNH2	1.778669	1.412141	2.24033	1.00E-06
HFE	0.252903	0.145464	0.439695	1.11E-06
DDX17	2.935689	1.902014	4.53113	1.16E-06
TAP2	0.419653	0.295044	0.59689	1.36E-06
IL11RA	3.183017	1.987917	5.096591	1.43E-06
APOD	1.233503	1.132303	1.343748	1.55E-06
CSRP1	1.467072	1.254286	1.715956	1.64E-06
LGR4	0.229825	0.12467	0.423674	2.45E-06

TAP1	0.617528	0.504297	0.756184	3.10E-06
PPP3R1	0.44518	0.316651	0.625879	3.23E-06
SDC2	1.491073	1.260032	1.764478	3.31E-06
VDR	0.423295	0.293965	0.609522	3.81E-06
PTGER3	2.236363	1.586377	3.152669	4.35E-06
FAM19A4	1.248233	1.13513	1.372606	4.75E-06
AKT3	1.937001	1.454575	2.579429	6.07E-06
EDNRA	1.526175	1.270711	1.832997	6.09E-06
TGFBR1	2.005086	1.482483	2.711916	6.32E-06
TGFB2	1.926372	1.448019	2.562749	6.74E-06
NFATC4	3.498278	2.025605	6.041626	7.06E-06
SEMA4C	2.292647	1.591604	3.302474	8.36E-06
FAM19A5	1.580548	1.291622	1.934105	8.81E-06
NR2F1	1.3617	1.188237	1.560486	8.97E-06
FGFR1	2.720608	1.745957	4.239343	9.75E-06
TOR2A	0.18655	0.08787	0.396048	1.24E-05
A2M	2.362291	1.597658	3.492875	1.65E-05
IL1R1	1.825173	1.386905	2.401936	1.75E-05
ANGPT1	1.616771	1.297136	2.01517	1.91E-05
PDIA3	0.532984	0.396479	0.716485	3.07E-05
FGF22	0.086557	0.027113	0.276333	3.60E-05
PIK3CA	1.911511	1.398399	2.612898	4.85E-05
CBLC	0.457021	0.312485	0.66841	5.42E-05
FGF1	4.283795	2.099677	8.739872	6.36E-05
CYR61	1.383119	1.177578	1.624536	7.77E-05
BCL10	0.482168	0.335666	0.692612	7.89E-05
FGF2	1.377027	1.174413	1.614597	8.16E-05
TNFRSF10A	0.61792	0.485467	0.786512	9.19E-05
TSLP	1.457079	1.206417	1.759821	9.30E-05

BACH2	1.602413	1.264844	2.030075	9.36E-05
GCGR	0.274145	0.142768	0.526416	0.000101
GREM2	1.381592	1.173258	1.626919	0.000106
VIM	1.511752	1.222671	1.86918	0.000135
RBP1	1.508866	1.221053	1.86452	0.000139
PMP2	1.959926	1.385664	2.772178	0.000143
PSMC3	0.465092	0.31347	0.690053	0.000143
KIR2DL3	0.253308	0.124733	0.514419	0.000145
OGN	1.132072	1.061801	1.206992	0.000148
R3HDML	0.217993	0.098868	0.480652	0.000159
COLEC12	1.265574	1.119705	1.430446	0.000164
IFIH1	0.5519	0.404872	0.752322	0.00017
KIR2DL1	0.305542	0.164662	0.566953	0.00017
RAC1	0.372939	0.22292	0.623918	0.000172
HLA.G	0.551	0.402941	0.753463	0.000189
IL17D	1.367162	1.15982	1.61157	0.000194
FGF7	1.640996	1.263643	2.131037	0.000203
PLXNA4	3.068033	1.693956	5.556713	0.000216
PLXND1	2.233715	1.454997	3.429206	0.000238
OBP2A	0.127498	0.042283	0.38445	0.000255
PPARG	0.772626	0.672447	0.887728	0.000272
NR2F2	1.494215	1.203191	1.855632	0.00028
SLC22A17	3.195511	1.706848	5.982542	0.000282
RARG	0.129229	0.042822	0.389983	0.000282
CTGF	1.407233	1.167801	1.695755	0.00033
CD81	1.959412	1.35709	2.829066	0.000332
GALR3	0.344135	0.192195	0.616194	0.000332
IL17RD	1.544755	1.21791	1.959315	0.000337
CXCL12	1.263258	1.11175	1.435413	0.000337

TGFBR3	1.291071	1.122558	1.484881	0.000344
KIR3DL1	0.236714	0.107391	0.521769	0.000353
HSPA2	1.271677	1.114604	1.450887	0.000353
PDGFRB	1.454448	1.183968	1.786719	0.000359
NPR1	2.67739	1.552952	4.615996	0.000394
IKBKE	0.440735	0.280082	0.693537	0.000397
KL	1.368425	1.150296	1.627917	0.000399
NRP2	1.976127	1.353925	2.884265	0.000415
HNF4G	0.739391	0.625087	0.874598	0.000425
VEGFB	2.012321	1.36192	2.973329	0.000447
CLEC11A	1.734986	1.274749	2.361389	0.000459
TFRC	0.414217	0.252519	0.679456	0.000482
TNC	1.283702	1.115431	1.477357	0.000494
FABP4	1.340109	1.136377	1.580367	0.000502
PAK3	1.297737	1.120266	1.503322	0.000514
AR	1.891736	1.318594	2.714003	0.000537
NOS2	0.614757	0.466634	0.809899	0.000542
GDF10	1.673232	1.249622	2.240442	0.000548
NCR1	0.189708	0.073778	0.487801	0.000561
LTBP2	1.340446	1.13405	1.584406	0.000594
BIRC5	0.711208	0.585351	0.864126	0.000604
HLA.C	0.553386	0.394005	0.77724	0.00064
ARTN	0.323391	0.167904	0.622865	0.000737
NEDD4	1.482913	1.179168	1.864899	0.000754
GHRHR	0.147105	0.048232	0.448659	0.000755
LIFR	1.304626	1.117456	1.523147	0.000764
PSMD13	0.457041	0.289666	0.72113	0.000765
GRP	1.215343	1.084422	1.362069	0.000798
CACYBP	0.525986	0.361228	0.765892	0.000805

CALR	0.428195	0.260149	0.704794	0.00085
CRHR1	0.161869	0.055409	0.472878	0.000871
PLTP	1.36036	1.134908	1.630599	0.000872
PAK1	0.479879	0.311224	0.739929	0.00089
CDH1	0.728113	0.603717	0.87814	0.000902
S1PR1	1.494901	1.178495	1.896256	0.000921
DEFB123	0.292468	0.141121	0.60613	0.000945
ANGPT4	0.240324	0.102367	0.564201	0.001059
TGFB3	2.019321	1.325793	3.075635	0.001062
CHUK	0.520121	0.350981	0.770771	0.001125
IFNA7	0.195249	0.072825	0.52348	0.001169
ANGPTL2	1.380501	1.134405	1.679984	0.001287
IGF1	1.356198	1.12593	1.633559	0.001331
S100A16	0.752348	0.631655	0.896104	0.001425
PML	0.309956	0.150778	0.637177	0.001443
HSP90AA1	0.511862	0.338878	0.773147	0.001459
FABP3	1.261872	1.092915	1.456948	0.001517
PSMD1	0.510904	0.337219	0.774046	0.001533
AVPR1A	1.966909	1.291947	2.994497	0.001608
IFNA14	0.294242	0.137482	0.629742	0.001626
SEMA3G	1.379393	1.12915	1.685095	0.001637
SPAG11A	0.277309	0.124706	0.61665	0.001657
PTK2B	0.255815	0.109354	0.598435	0.001666
ILK	1.72517	1.224946	2.429669	0.001801
PSME3	0.544933	0.371729	0.798841	0.001866
TMSB15B	2.337231	1.365785	3.999639	0.001953
NRP1	1.807961	1.242598	2.630554	0.001967
AGTR2	1.86907	1.257132	2.778883	0.001996
ELN	2.903888	1.475877	5.713597	0.00202

PDYN	0.286057	0.12916	0.633542	0.002035
HDAC1	0.533935	0.357797	0.796783	0.002125
PDGFRL	1.211584	1.071627	1.369821	0.00218
NFKBIB	0.384253	0.20826	0.708971	0.002209
NDP	1.27476	1.090971	1.489512	0.002243
CASP3	0.634952	0.474476	0.849705	0.002246
DMBT1	0.888048	0.822896	0.958359	0.002258
F2RL1	0.77392	0.655749	0.913387	0.002432
PDGFC	1.315135	1.101602	1.570059	0.002442
IL32	0.74465	0.615005	0.901625	0.002519
IL1F10	0.326438	0.157184	0.677945	0.002679
HLA.B	0.644954	0.483434	0.860441	0.002864
THBS1	1.458024	1.137301	1.869191	0.00293
ANXA6	1.796834	1.220941	2.644363	0.002954
HLA.F	0.65894	0.500394	0.86772	0.002975
MASP2	0.10657	0.024298	0.467415	0.002995
OGFR	0.292439	0.1294	0.6609	0.003121
CRIM1	1.464362	1.136473	1.88685	0.003187
RBP7	1.277494	1.085393	1.503593	0.003224
GMFB	0.580894	0.404468	0.834275	0.003272
FGF13	1.158379	1.049934	1.278025	0.003373
FGF9	1.242038	1.074314	1.435947	0.003407
IFNA17	0.407615	0.223193	0.744421	0.003495
SHC2	1.522886	1.148175	2.019884	0.003513
TAPBP	0.527611	0.342454	0.81288	0.003739
FGF17	0.247448	0.095777	0.639301	0.00393
SEMA4G	0.567687	0.385352	0.836296	0.004178
ROBO1	1.274758	1.079265	1.505662	0.004263
IGF1R	1.398728	1.111105	1.760806	0.004277

PRLR	0.60599	0.428856	0.856286	0.004519
CORT	0.410052	0.221418	0.759391	0.004577
HLA.A	0.569553	0.383411	0.846065	0.005306
LCN15	0.394186	0.203822	0.762347	0.00567
TRIM5	0.600985	0.417106	0.865928	0.006286
IL27	0.381476	0.190912	0.762256	0.00636
OAS1	0.799683	0.680641	0.939545	0.006563
SECTM1	0.722319	0.57109	0.913595	0.006649
MAP2K2	0.392564	0.199654	0.771869	0.006716
PSPN	0.346271	0.160424	0.747417	0.006901
MET	0.623012	0.440805	0.880533	0.007346
VEGFC	1.299997	1.072638	1.575549	0.007476
MC4R	0.355021	0.166219	0.758275	0.007481
AMELX	0.208244	0.065884	0.658212	0.007534
HGF	1.412606	1.095986	1.820693	0.007635
S100A14	0.870131	0.785459	0.96393	0.007738
ULBP1	0.279389	0.108877	0.716939	0.008001
GPHB5	0.308175	0.128039	0.74174	0.008624
MC2R	0.180194	0.049982	0.649633	0.008813
PSMD14	0.566394	0.369891	0.86729	0.008925
CCL20	0.879932	0.799178	0.968845	0.009203
OPRL1	0.287756	0.112389	0.736757	0.009408
OBP2B	0.420661	0.218604	0.809483	0.009519
CNTF	0.31262	0.129679	0.753641	0.009599
CSHL1	0.144449	0.033073	0.630894	0.010101
ESRRA	0.547104	0.344186	0.869656	0.010755
UCN3	0.42207	0.217524	0.81896	0.010757
BMPR2	1.821914	1.146347	2.895606	0.011157
XCR1	0.35239	0.157144	0.790223	0.011362

TCF7L2	0.627815	0.437118	0.901708	0.011733
NOX4	1.323595	1.064109	1.646358	0.0118
CER1	0.360461	0.16224	0.800864	0.012239
PSMC4	0.627516	0.435624	0.903935	0.012338
PTHLH	1.287169	1.055194	1.570142	0.01278
CMTM3	1.673557	1.114803	2.512365	0.012982
NRAS	0.62789	0.433782	0.908858	0.013646
OASL	0.812327	0.688634	0.958238	0.013659
NR4A3	1.337428	1.061195	1.685567	0.013772
TIE1	1.676437	1.111314	2.528935	0.013773
PTX3	1.14265	1.027413	1.270813	0.013949
HSPA4	0.63423	0.439537	0.915162	0.01494
SEMA6A	1.336933	1.056814	1.6913	0.015495
TNFRSF10B	0.712125	0.540353	0.9385	0.015924
SCG2	1.141101	1.024177	1.271373	0.016707
IL17RC	0.28347	0.100827	0.796963	0.016836
SEMA4B	0.726409	0.558591	0.944646	0.017087
CCL25	0.845919	0.737078	0.970831	0.017255
LEFTY1	0.920171	0.858904	0.985807	0.017953
GREM1	1.109008	1.017165	1.209143	0.018985
ENG	1.612557	1.080824	2.405887	0.019247
AZU1	0.414449	0.198215	0.866575	0.019258
AVPR2	0.350374	0.145498	0.843737	0.019341
GPI	0.726185	0.555092	0.950012	0.019593
CMTM5	1.985358	1.113231	3.540727	0.02016
GHRH	0.472886	0.250541	0.892554	0.020851
PPP4C	0.624115	0.417797	0.932317	0.021323
NPPB	0.452347	0.230076	0.889348	0.021451
PSMD3	0.73287	0.562302	0.955178	0.021492

PSMC6	0.545468	0.323504	0.919726	0.022972
GNAI1	1.195968	1.024988	1.395469	0.022995
SEMA3E	1.221247	1.026963	1.452286	0.023764
CMA1	1.410472	1.04599	1.901959	0.024149
SCTR	2.138981	1.103668	4.145487	0.024314
PGF	1.460959	1.049524	2.033685	0.024679
VIPR2	2.562536	1.1232	5.846323	0.025349
SRC	0.316941	0.114653	0.876132	0.026769
PRKCG	0.269298	0.083732	0.866111	0.027727
MCHR2	0.368052	0.150052	0.902769	0.029006
PLSCR1	0.642267	0.431558	0.955856	0.029071
ESRRB	0.29938	0.101223	0.885453	0.029269
CCL16	2.198284	1.079693	4.475768	0.029906
CMTM1	0.453106	0.221451	0.927088	0.030218
VAV3	0.839607	0.715599	0.985105	0.032033
RETNLB	0.339894	0.126381	0.914127	0.03253
TSHR	0.322457	0.114012	0.911999	0.032874
DCD	0.429483	0.197123	0.935738	0.033409
BID	0.754256	0.580457	0.980093	0.034821
PIK3CB	0.676273	0.470271	0.972514	0.03483
AVPR1B	0.454917	0.217413	0.951872	0.036537
PTH2	0.431322	0.195557	0.951328	0.037197
PAK4	0.43841	0.20175	0.952677	0.037307
AMBN	0.476619	0.236912	0.958863	0.037733
PYY	0.465309	0.224943	0.962522	0.039116
CYSLTR2	0.470721	0.229404	0.965885	0.039916
CNTFR	0.456271	0.215717	0.965075	0.040075
FGF14	1.533183	1.017007	2.311342	0.041302
CCL1	0.44878	0.207618	0.970068	0.041627

HLA.E	0.718433	0.521286	0.990141	0.043331
HTR3A	1.36454	1.007377	1.848335	0.044704
DEFB127	0.302742	0.093866	0.976415	0.04551
PGLYRP4	0.541881	0.296421	0.9906	0.04652
EDN2	0.5054	0.257872	0.990526	0.046846
LCN8	0.364863	0.134853	0.987191	0.047106
TSHB	0.42658	0.182839	0.995249	0.048726

Abbreviations: IRGs: immune-related genes; HR: hazard ratio; CI: confidence interval.

Table S2 GO analysis results of 266 survival related genes

Ontology	ID	Description	p.adjust	Gene ID
Biological process	GO:0050920	regulation of chemotaxis	3.88E-22	ARTN/AZU1/BMPR2/CALR/CCL1/CXCL12/
Biological process	GO:0001667	ameboidal-type cell migration	4.57E-19	AGTR2/AKT3/ANGPT1/ANGPT4/ANXA6/BMPR2/CALR/CER1/FGF1/FGF2/FGF7/FGF
Biological process	GO:0060326	cell chemotaxis	3.39E-18	AGTR1/AZU1/CALR/CCL1/CCL16/CCL20/CCL25/CXCL12/EDN2/F2RL1/FGF1/FGF2/F
Biological process	GO:0050921	positive regulation of chemotaxis	1.15E-16	ARTN/AZU1/BMPR2/CALR/CCL1/CXCL12/
Biological process	GO:0061138	morphogenesis of a branching epithelium	3.85E-15	AGTR2/AR/EDNRA/ENG/FGF1/FGF2/FGF7/FGFR1/GREM1/HGF/ILK/LGR4/MET/NFAT
Biological process	GO:0051897	positive regulation of protein kinase B signaling	1.25E-14	ANGPT1/ENG/FGF1/FGF17/FGF2/FGF22/FGF7/FGF9/FGFR1/HGF/HSP90AA1/IGF1R/I
Biological process	GO:0001763	morphogenesis of a branching structure	1.60E-14	AGTR2/AR/EDNRA/ENG/FGF1/FGF2/FGF7/FGFR1/GREM1/HGF/ILK/LGR4/MET/NFAT
Biological process	GO:2000027	regulation of animal organ morphogenesis	4.12E-14	AGTR2/AMELX/AR/BMPR2/CNTF/ENG/FGF1/FGF7/FGFR1/GREM1/HGF/LGR4/PSMB
Biological process	GO:0048754	branching morphogenesis of an epithelial tube	6.23E-14	AGTR2/AR/EDNRA/ENG/FGF1/FGF2/GREM1/ILK/LGR4/MET/NFATC4/NRP1/PAK1/PG
Biological process	GO:0060485	mesenchyme development	1.07E-13	AMELX/ANXA6/BMPR2/CER1/DDX17/EDNRA/ENG/FGF9/FGFR1/GREM1/HGF/IL17R

Ontology	ID	Description	p.adjust	Gene ID
Cellular component	GO:0062023	collagen-containing extracellular matrix	6.72E-08	A2M/AMELX/ANGPT1/ANGPT4/ANGPTL1/
Cellular component	GO:0022624	proteasome accessory complex	6.72E-08	PSMC3/PSMC4/PSMC6/PSMD1/PSMD13/PSMD14/PSMD3/PSME3
Cellular component	GO:0005838	proteasome regulatory particle	5.75E-07	PSMC3/PSMC4/PSMC6/PSMD1/PSMD13/PSMD14/PSMD3
Cellular component	GO:0034774	secretory granule lumen	3.18E-06	A2M/AZU1/GPI/GRP/HGF/HSP90AA1/IGF1/
Cellular component	GO:0060205	cytoplasmic vesicle lumen	3.18E-06	A2M/AZU1/GPI/GRP/HGF/HSP90AA1/IGF1/PENK/PSMC3/PSMD1/PSMD13/PSMD14/P
Cellular component	GO:0031983	vesicle lumen	3.18E-06	A2M/AZU1/GPI/GRP/HGF/HSP90AA1/IGF1/PENK/PSMC3/PSMD1/PSMD13/PSMD14/P
Cellular component	GO:0000502	proteasome complex	5.05E-06	PSMB8/PSMC3/PSMC4/PSMC6/PSMD1/PSMD13/PSMD14/PSMD3/PSME3
Cellular component	GO:1905369	endopeptidase complex	9.89E-06	PSMB8/PSMC3/PSMC4/PSMC6/PSMD1/PSMD13/PSMD14/PSMD3/PSME3
Cellular component	GO:0031093	platelet alpha granule lumen	8.18E-05	A2M/HGF/IGF1/TGFB2/TGFB3/THBS1/VEGFB/VEGFC
Cellular component	GO:1905368	peptidase complex	9.36E-05	PSMB8/PSMC3/PSMC4/PSMC6/PSMD1/PSMD13/PSMD14/PSMD3/PSME3

Ontology	ID	Description	p.adjust	Gene ID
Molecular function	GO:0030546	signaling receptor activator activity	1.41E-53	AMBN/AMELX/ANGPT4/ARTN/CCL1/CCL1
Molecular function	GO:0048018	receptor ligand activity	5.77E-53	AMBN/AMELX/ARTN/CCL1/CCL16/CCL20/ CCL25/CER1/CLEC11A/CMTM1/CMTM3/C
Molecular function	GO:0008083	growth factor activity	1.67E-24	AMBN/AMELX/ARTN/CLEC11A/CNTF/CSH L1/CXCL12/FGF1/FGF13/FGF14/FGF17/FG
Molecular function	GO:0005125	cytokine activity	1.79E-18	CCL1/CCL16/CCL20/CCL25/CER1/CMTM1/
Molecular function	GO:0008528	G protein-coupled peptide receptor activity	3.90E-17	AGTR1/AGTR2/AVPR1A/AVPR1B/AVPR2/C RHR1/CYSLTR2/EDNRA/F2RL1/GALR3/GC
Molecular function	GO:0001653	peptide receptor activity	6.20E-17	AGTR1/AGTR2/AVPR1A/AVPR1B/AVPR2/C RHR1/CYSLTR2/EDNRA/F2RL1/GALR3/GC
Molecular function	GO:0070851	growth factor receptor binding	1.37E-16	ARTN/CBLC/CNTF/FGF1/FGF17/FGF2/FGF 22/FGF7/FGF9/GREM1/IL1F10/IL1R1/KL/P
Molecular function	GO:0019955	cytokine binding	6.91E-16	A2M/BMPR2/CER1/CNTFR/ENG/FGF2/GH R/GREM1/GREM2/IL11RA/IL1R1/LIFR/LTB
Molecular function	GO:0019838	growth factor binding	7.24E-16	A2M/BMPR2/CRIM1/ENG/FGFR1/GHR/GH RHR/IGF1R/IL11RA/IL1R1/KL/LIFR/LTBP1/L
Molecular function	GO:0005126	cytokine receptor binding	4.19E-15	BID/CASP3/CCL1/CCL16/CCL20/CCL25/C NTF/CSHL1/CXCL12/ENG/GREM1/IFNA14/

The top 10 GO terms are listed. **Abbreviations:** GO: Gene Ontology.

Table S3 KEGG pathway analysis results of 266 survival related genes

ID	Description	p.adjust	Gene ID
hsa05218	Melanoma	5.96E-15	AKT3/CDH1/FGF1/FGF13/FGF14/FGF17/FGF2/FGF22/FGF7/FGF9/FGFR1/HGF/IGF1/IGF1R/MAP2K2/MET/NRAS/PDG
hsa05200	Pathways in cancer	5.96E-15	AKT3/AR/BID/BIRC5/CASP3/CBLC/CDH1/CHUK/FGF1/FGF
hsa04650	Natural killer cell mediated cytotoxicity	3.79E-13	BID/CASP3/IFNA14/IFNA17/IFNA7/KIR2DL1/KIR2DL3/KIR3DL1/MAP2K2/NCR1/NFATC4/NRAS/PAK1/PIK3CA/PIK3CB/
hsa04060	Cytokine-cytokine receptor interaction	8.68E-12	BMPR1B/BMPR2/CCL1/CCL16/CCL20/CCL25/CNTF/CNTFR/CXCL12/GHR/HGF/IFNA14/IFNA17/IFNA7/IL11RA/IL1R1/I
hsa05211	Renal cell carcinoma	6.20E-10	AKT3/HGF/MAP2K2/MET/NRAS/PAK1/PAK3/PAK4/PGF/PIK
hsa04510	Focal adhesion	8.91E-09	AKT3/HGF/IGF1/IGF1R/ILK/MET/PAK1/PAK3/PAK4/PDGFC/
hsa04360	Axon guidance	2.06E-08	CXCL12/GNAI1/MET/NFATC4/NRAS/NRP1/PAK1/PAK3/PAK
hsa04010	MAPK signaling pathway	5.45E-07	AKT3/CASP3/CHUK/FGF1/FGF13/FGF14/FGF17/FGF2/FG
hsa05220	Chronic myeloid leukemia	6.90E-07	AKT3/CBLC/CHUK/HDAC1/MAP2K2/NRAS/PIK3CA/PIK3CB
hsa04662	B cell receptor signaling pathway	8.69E-07	AKT3/BCL10/CD81/CHUK/MAP2K2/NFATC4/NFKBIB/NRAS/PIK3CA/PIK3CB/PPP3R1/RAC1/VAV3

The top 10 KEGG terms are listed. **Abbreviations:** KEGG: Kyoto Encyclopedia of Genes and Genomes.

Table S4 Pearson correlation analysis results of the relationships between 22 immune infiltrating cells and OS

Immune infiltrating cells	r	P-value
T cells CD4 memory activated	0.162399	0.004805
Plasma cells	0.132192	0.022014
Macrophages M2	-0.12379	0.032085
NK cells resting	0.114339	0.047858
T cells gamma delta	-0.10223	0.077085
Monocytes	0.088887	0.124495
T cells CD4 memory resting	-0.08267	0.153172
Mast cells activated	-0.0785	0.17507
NK cells activated	-0.06325	0.274814
T cells regulatory Tregs	0.061997	0.284456
Dendritic cells activated	0.061256	0.290261
Macrophages M1	0.060935	0.292802
T cells follicular helper	0.050943	0.379272
T cells CD4 naive	0.04753	0.412065
Neutrophils	0.04678	0.419487
Mast cells resting	-0.03422	0.554872
Macrophages M0	0.023241	0.688474
B cells naive	-0.01534	0.791263
Dendritic cells resting	0.015142	0.793945
Eosinophils	0.010163	0.860841
T cells CD8	-0.00814	0.888364
B cells memory	0.007232	0.900729

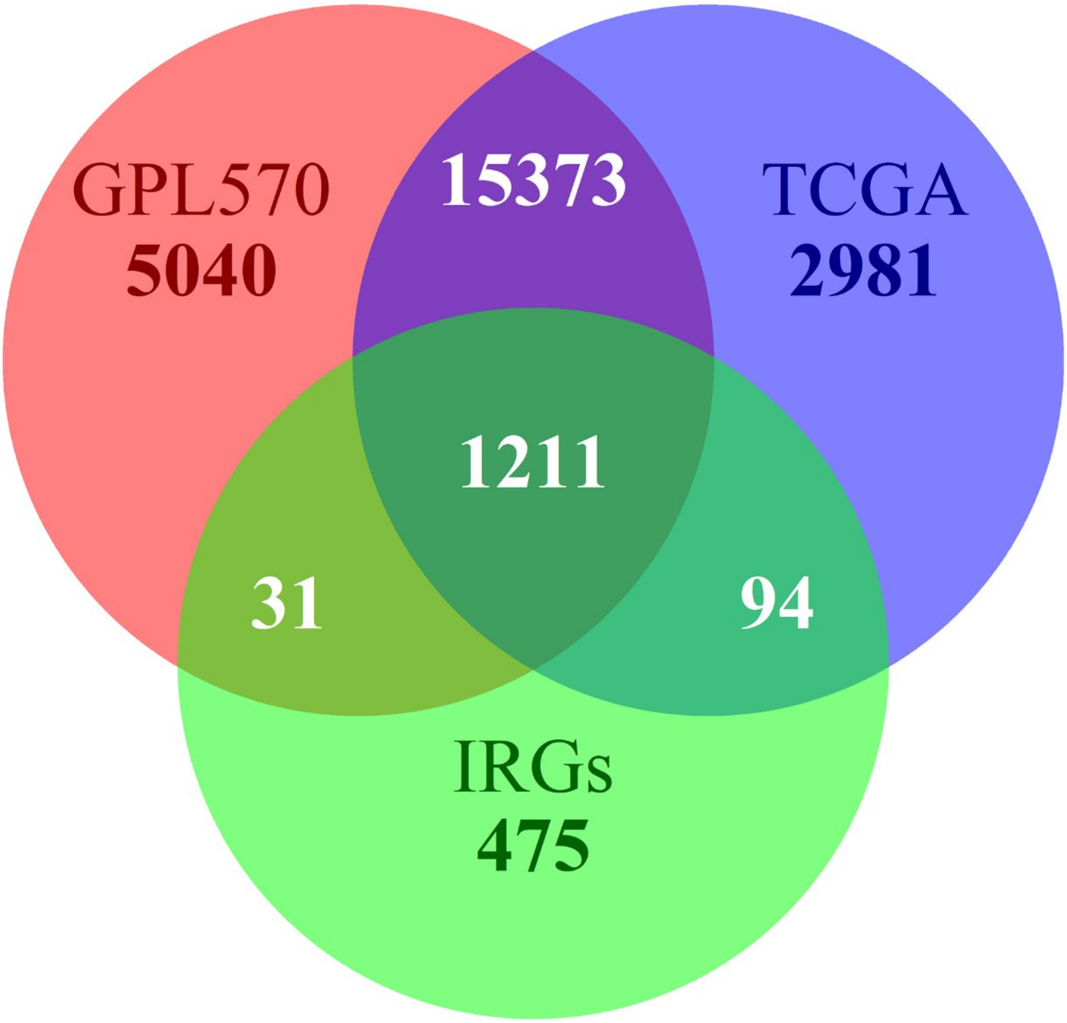

Figure S1 Venn diagram indicating overlapped IRGs from GEO database, TCGA database and ImmPort database.

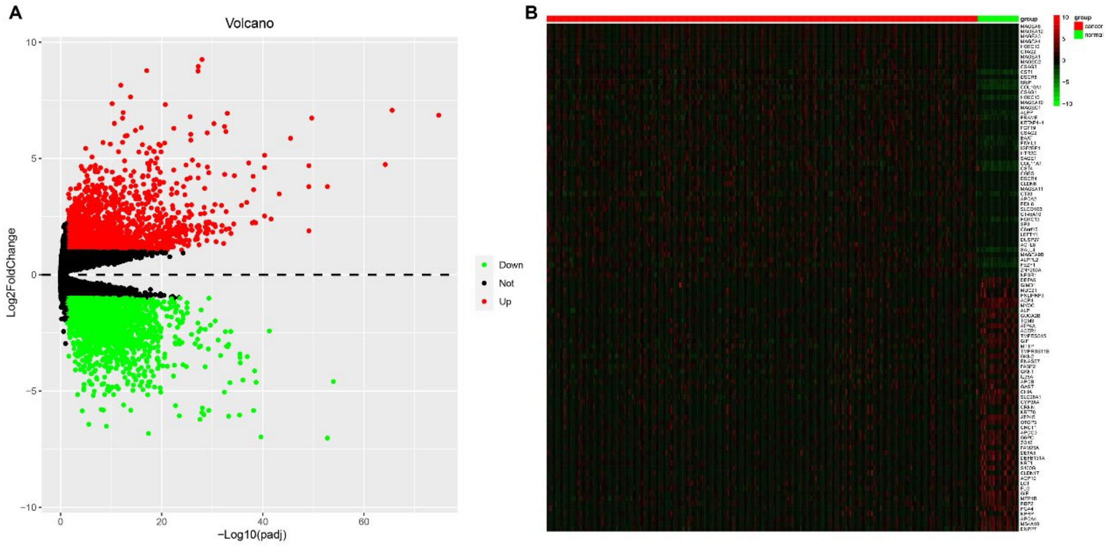

Figure S2 Visualization of differentially expressed genes via volcano plot and heatmap. (A) Volcano plot of the DEGs in TCGA-STAD. Red dots represent up-regulated genes, green dots represent down-regulated genes and dark dots represent genes with no significance. (B) Heatmap of top 50 up-regulated and top 50 down-regulated DEGs in TCGA-STAD.

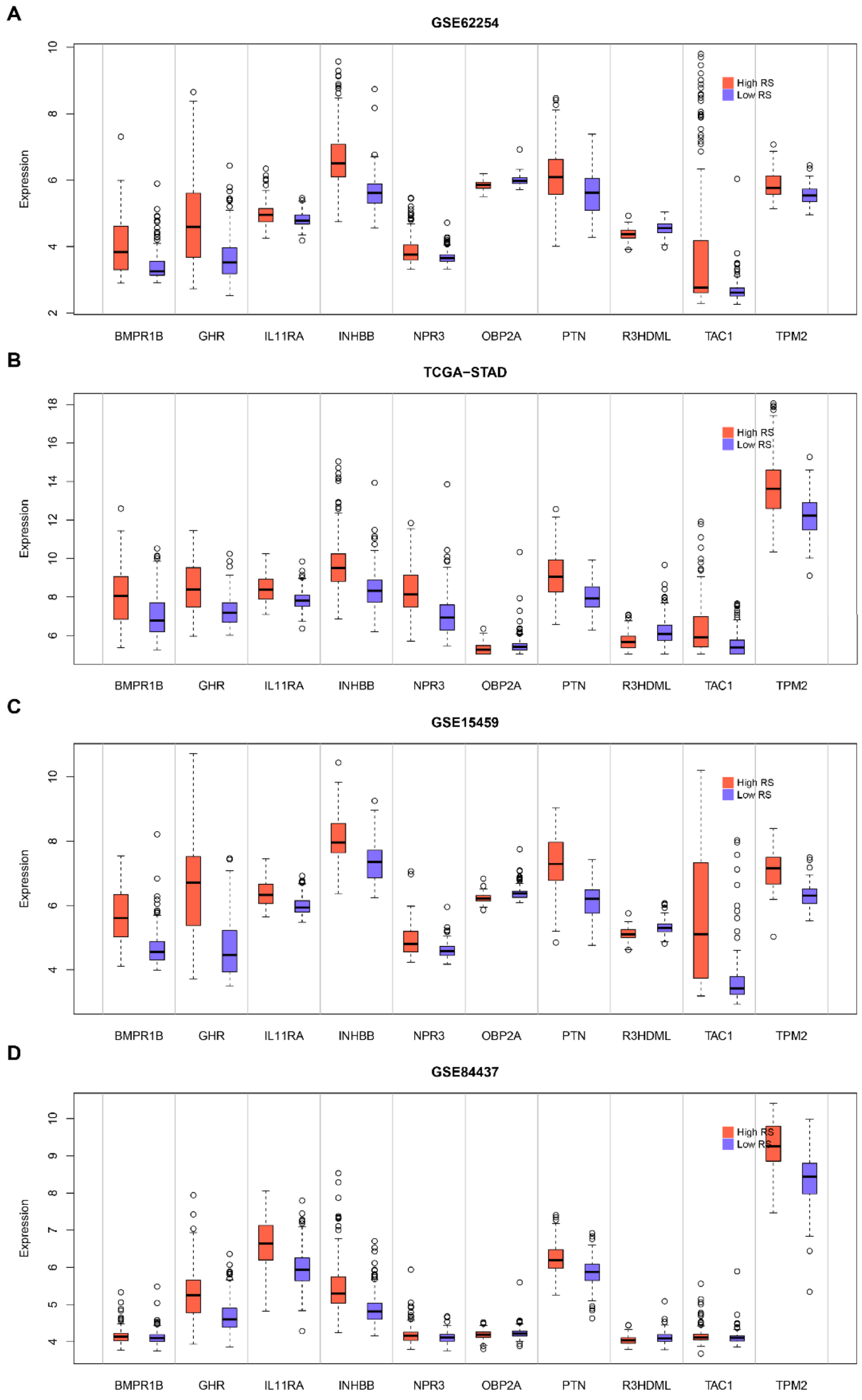

Figure S3 Expression profiles of 10 IRGs in high RS and low RS groups in the training dataset (A),the TCGA dataset (B), the validation dataset Ⅰ (C) and the validation dataset Ⅱ (D). The 8 risk factors were higher in the high RS group and the 2 protective factors were higher in the low RS group.

Abbreviations: RS: risk score.

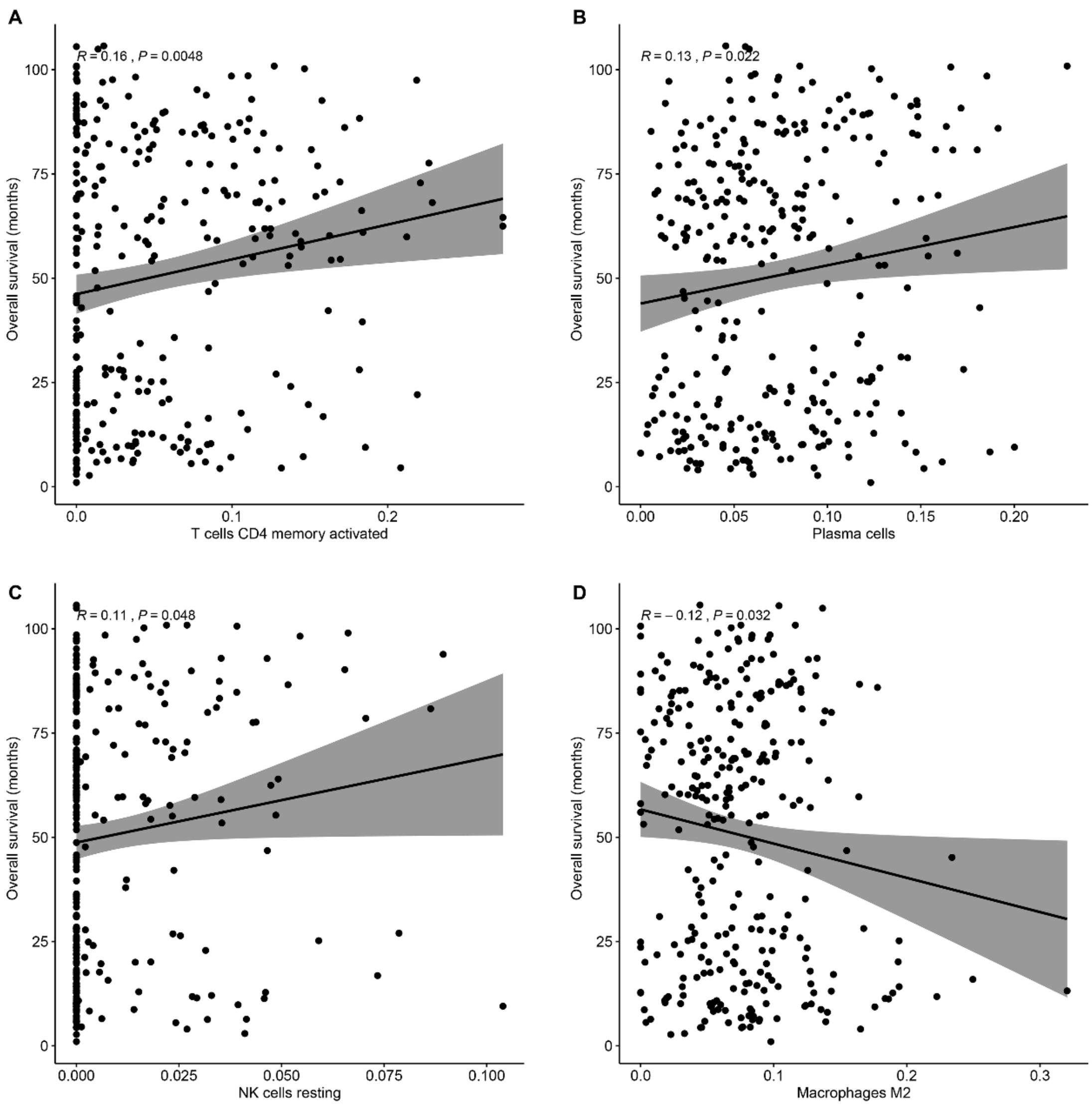

Figure S4 Correlation plots of the relationships between immune infiltrating cells and OS. (A) T cells CD4 memory activated. (B) Plasma cells. (C) NK cells resting. (D)Macrophages M2.
